# The Role of Meeting Exercise and Nutrition Guidelines on Sleep during Pregnancy

**DOI:** 10.3390/nu15194213

**Published:** 2023-09-29

**Authors:** Traci A. McCarthy, Sarah M. Velez, Jennifer F. Buckman, Andrea M. Spaeth

**Affiliations:** 1 Department of Exercise Science, School of Health Sciences, Stockton University, Galloway, NJ 08205, USA; 2 Department of Kinesiology and Applied Physiology, School of Arts and Sciences, Rutgers University, New Brunswick, NJ 08901, USA; sarahmvelez@gmail.com (S.M.V.); jbuckman@rutgers.edu (J.F.B.); ams853@kines.rutgers.edu (A.M.S.)

**Keywords:** perinatal, exercise, nutrition, sleep, women’s health

## Abstract

Sleep disturbances are common during pregnancy. This study determined whether meeting physical activity or dietary guidelines during pregnancy was associated with improved sleep. Third trimester pregnant women (n = 49, 31.9 ± 4.1 years) completed physical activity and sleep questionnaires and then wore a wrist actigraph 24 h/day and completed three 24 h dietary recalls across two weeks. Participants who reported meeting physical activity guidelines (>150 min moderate-to-vigorous physical activity [MVPA]/week, n = 23) or dietary guidelines (≥1.1 g protein/kg body weight/day, n = 26 or ≥25 g fiber/day, n = 16) were compared to those who were physically inactive (<90 min/week) or did not meet dietary guidelines, respectively. Multivariate ANOVAs and Mann–Whitney U tests compared groups and correlations were conducted between physical activity, diet, and sleep variables. Physical activity groups did not differ in objective sleep measures (*p*s > 0.05); however, the active group reported better sleep quality (*p* = 0.049). Those who met protein guidelines exhibited longer sleep duration and less wake-after-sleep-onset (*ps* < 0.05). Across all participants, higher weekly MET mins/week of MVPA associated with better sleep quality (*p* = 0.02), and a diet higher in fat and lower in carbohydrates associated with longer sleep duration (*p*s < 0.05). Meeting physical activity and nutrition guidelines positively associates with improved sleep, with protein associated with objective measures and physical activity with subjective measures.

## 1. Introduction

A host of physical and physical changes occur throughout pregnancy including anatomical, breathing, metabolic, and hormone fluctuations [1]. These changes can cause back pain, restless leg syndrome, gastroesophageal reflux, frequent urination, and uterine contractions, all of which can impact sleep [2,3,4]. Sleep disturbances, daytime sleepiness, and fatigue are among the top complaints women have during pregnancy [5]. Sleep duration decreases as pregnancy progresses, with the prevalence of short sleep duration (<7 h) increasing from 26% in the first trimester to 40% in the third trimester [6]. In the third trimester, decreases in both rapid eye movement (REM) sleep and slow wave sleep (SWS) have been observed and wake after sleep onset (WASO) is particularly evident [7]. Women with shorter habitual sleep durations and greater sleep disturbances during pregnancy are at increased risk for anxiety and depression [8], placental abruption [9], cesarean section [10], placental abruption and preterm delivery [9], and postpartum weight gain [11]. Thus, treatments targeting the improvement of sleep during pregnancy are needed.

Pharmacological treatments are often used to improve sleep in the general adult population; however, many sleep-related drugs are contraindicated by the Food and Drug Administration (FDA) during pregnancy [12]. Moreover, pregnant women are more hesitant to take medication regardless of whether it has been FDA approved. An observational study found women ascribed more risk to all types of drugs [13], suggesting that it is critical to investigate non-pharmacological alternatives for improving sleep in pregnancy. Thus, continued research into implementing guidelines and interventions related to lifestyle behaviors is needed. Common non-pharmacological treatments and lifestyle changes include meditation, cognitive behavioral therapy, maintaining a consistent sleep routine, caffeine avoidance, and limiting screen exposure prior to sleep [14]. A growing body of evidence further suggests that exercise and certain dietary factors are associated with improved sleep duration and quality in adults, implying that exercise or dietary interventions specifically tailored to pregnant women may also have significant public health value [15,16].

A 2023 systematic review and meta-analysis found that exercise interventions during pregnancy improved subjective sleep quality, sleep disturbances, and insomnia severity; however, there were a limited number of controlled trials available and substantial methodological differences across interventions [17]. One well-designed aquatic intervention involved three 60 min sessions per week during pregnancy and observed improvements in subjective measures of sleep quality, sleep latency, sleep duration, and sleep efficiency compared to the control group [18]. Similarly, an observational study found that women who reported higher physical activity scores, which reflected duration (cumulative minutes per week) and intensity (light vs. moderate vs. vigorous), exhibited better reported sleep quality than women with lower activity scores [19]. The American College of Obstetricians and Gynecologists (ACOG) recommends pregnant women perform moderate intensity physical activity for a minimum of 150 min per week [20,21]. Despite evidence supporting the benefits of exercise, physical activity levels decrease by 42% compared to pre-pregnancy levels and only about 19% of pregnant women meet the ACOG physical activity guidelines [22].

Dietary factors such as macronutrient composition and daily caloric intake can also impact sleep in adults [23,24,25,26]. Higher fiber, protein, and fat intake have been associated with improved sleep quality, specifically, more slow wave sleep and rapid eye movement sleep [24,27], whereas hyperphagia and increased carbohydrate consumption have been associated with poorer sleep outcomes [28]. During pregnancy, women who were maintaining vegetarian diets or diets high in carbohydrates reported poorer sleep quality [29,30] whereas women who were consuming a diet high in fruits and vegetables, with less red meat, reported better sleep quality [31,32,33]. Both higher monounsaturated fat consumption, supplemental iron, and supplemental folic acid during pregnancy have been associated with better sleep quality during pregnancy [29,30]. A recent meta-analysis highlighted the needed for more work in this area, as there are a very limited number of high-quality studies examining the relationships between dietary factors and sleep during pregnancy [16].

In pregnancy, caloric demand increases to support the growth of the mother and fetus by an average of 300 kcal/day with a maximum of +452 kcal/day in the third trimester [34]. Protein recommendations increase from 0.8 g of protein per kg body weight per day pre-pregnancy to 1.1g protein/kg body weight/day during pregnancy [34]. Fiber which is derived from fruit, vegetables, legumes, and whole grains, reflects a high-quality diet and is associated with lower risk of eclampsia, excessive gestational weight gain, gestational diabetes mellitus, and constipation [35,36]. It is recommended by the American Pregnancy Association that pregnant women consume at least 25–28 g of fiber per day. Meeting fiber and protein guidelines may be particularly important for sleep given prior work on these nutrients and sleep in non-pregnant adults [23,26]. The effect of meeting dietary guidelines for protein and fiber during pregnancy on sleep has not been thoroughly investigated.

The current study addresses these gaps by comparing objective and subjective sleep measures between pregnant women who are or are not physically active and who do or do not consume the recommended amount of protein or fiber per day [37]. It is hypothesized that women who meet current physical activity or dietary guidelines during pregnancy will exhibit improved sleep (less wake-after-sleep-onset, longer sleep duration, higher subjective sleep quality) and less daytime sleepiness compared to those who do not. Additionally, correlations between physical activity, dietary factors, and sleep were examined.

## 2. Materials and Methods

This study was reviewed for ethical compliance by the Rutgers University Institutional Review Board (Pro2021000624). All participants provided informed written consent prior to participation.

- Participants

Participants were recruited in their second trimester (between gestation weeks 20–28). Study posters and brochures were posted in maternal health clinics, community centers, shopping centers, fitness facilities, and common gathering areas in the greater New Brunswick community. Social medial posts were advertised on Twitter, Facebook, and Instagram. Recruitment was also performed at community events in Middlesex and Monmouth Counties in New Jersey. Interested women were screened for eligibility using the study’s inclusion/exclusion criteria. Inclusion/exclusion criteria were implemented to identify participants eligible for a larger parent study. Participants were included if they (1) were between 12–28 weeks pregnant; (2) were between the ages of 18–40 y to minimize include women considered at higher risk of pregnancy related complications due to age; (3) could understand and complete all study procedures and measures; (4) reported either physical inactivity (<90 min of exercise per week) [38] or meeting ACSM’s physical activity guidelines (>150 min of moderate-level physical activity per week) [39]; (5) were willing to complete all study procedures, commit to two weeks of travel-free time, and adhere to their typical sleep and activity routine; (6) obtained medical clearance for participation in the study from their physician (PARmed-X for pregnancy); and (7) had a negative gestation diabetes mellitus test. Participants were excluded if they (1) had a pre-pregnancy history of severe low back pain or back surgery; (2) had a diagnosed sleep disorder; (3) had any contraindication to participating in moderate physical activity including but not limited to severe anemia, maternal cardiac dysrhythmia, chronic bronchitis, poorly controlled Type 1 or Type 2 diabetes mellitus, poorly controlled hypertension, heart disease, and restrictive lung disease; (4) had a non-singleton pregnancy; (5) had an orthopedic and/or cardiovascular limitation; (6) had low body weight (BMI < 18) or extreme obesity (BMI > 40) pre-pregnancy; or (7) experienced one or more of the following during pregnancy: premature labor, placenta previa, poor fetal growth, premature rupture of membranes, preeclampsia, uterine growth retardation, incompetent cervix, persistent vaginal bleeding, anemia, or gestational diabetes.

- Protocol

Participants came to the research center between 28–32 weeks gestation and were further educated about the study, screened for participation, and underwent informed consent. Once consent was obtained, participants completed a series of questionnaires including a demographic questionnaire, the pregnancy physical activity questionnaire (PPAQ) [40], the Pittsburg Sleep Quality Index (PSQI) [41], and the Epworth Sleepiness Scale (ESS) [41]. After completing questionnaires, participants performed a physical exam including height, weight, resting heart rate, blood pressure, and oxygen saturation.

Participants were educated on how to use the wrist actigraph (Philips Actiwatch) and heart rate monitor (Polar Verity Sense) and instructed to wear the actigraph 24 h a day for two consecutive weeks and to wear the heart rate monitor during voluntary physical activity. Participants were also asked to complete a sleep diary every morning and evening electronically. During the two weeks of home sleep and activity monitoring, participants were randomly sent 24 h dietary recalls (two on weekdays, one on a weekend day) to complete online using the Automated Self-Administered 24 h (ASA24) Dietary Assessment Tool, version (2020), developed by the National Cancer Institute, Bethesda, MD [42]. Upon completion of the study, participants were compensated financially for their time.

- Measures

To account for potential covariates, participant age, parity, gestational age, race, marital status, gestational weight gain, and socioeconomic status were collected via the demographic questionnaire.

The PPAQ was used to group participants by physical activity. This questionnaire estimates the amount of time an individual spends in a range of household, caregiving, occupational, and exercise activities during their current trimester to provide an estimate of their average weekly energy expenditure (MET-h·week^−1^) along with category value (light, moderate, or vigorous) physical activity METs. Quantitative information from the intensity and duration responses were used to determine the amount of moderate to vigorous intensity physical activity (MVPA) an individual performs [40]. Individuals who reported performing ≥150 min of MVPA or more per week were included in the physically active (PA) group. Individuals who reported <90 min of MVPA per week were included in the not physically active group (NPA). Information collected from daily diaries and heart rate monitors was used to support group classifications.

The Pittsburg Sleep Quality Index (PSQI) evaluates subjective sleep quality as a global score over the past month. The questionnaire asks participants to freely answer questions about their sleep duration, sleep quality, and sleep latency and then asks how often participants experience a variety of issues related to their sleep in the past 30 days and how severe various sleep issues have been using a 4-point Likert scale. The global score is comprised of several components: sleep quality, sleep latency, sleep duration, sleep efficiency, sleep disturbances, use of sleeping medication, and daytime dysfunction [41,43]. Higher scores indicate poorer sleep outcomes and a score of >5 is considered clinically meaningful. The Epworth Sleepiness Scale lists eight daytime activities (e.g., watching TV, as a passenger in a car) and participants are asked to rate the likelihood of dozing using a 4-point scale (0–3). Scores are summed to determine daytime sleepiness with higher scores indicating greater daytime sleepiness. This scale has shown good reliability validity [41]. Individuals with scores ≥10 are considered clinically meaningful [41].

A twice-daily diary was completed to provide a subjective daily measure of sleep and exercise. Sleep questions covered whether the participant woke up during the night, for how long, and for what reason (pain, urination, fetal movement) as well as the time they attempted to fall asleep, the time they woke up, and the time they got out of bed in the morning. Exercise questions included whether the participant exercised, at what time, for how long, and at what intensity.

Sleep was assessed objectively using continuous monitoring across two weeks using the Actiwatch Spectrum Plus (Phillips Respironics, Murrysville, PA) worn on the non-dominant wrist. Wrist actigraphy is a widely used, reliable and valid method of sleep measurement [44]. It monitors 24 h activity through recordings of 1 min epochs and determines sleep/wake based on movement [45]. Sleep variables were sleep onset (bedtime), sleep offset (wake time), sleep period (time from sleep onset to sleep offset), wake-after-sleep-onset (epochs scored as wake during the sleep period), total sleep time (epochs scored as sleep during the sleep period), sleep efficiency (total sleep time/sleep period × 100), and sleep fragmentation index (a measure of short sleep bouts [≤1 min] and movement during sleep). The epochs are scored using the Actiware 6.0 software and data were processed using the 3/5 method [46].

Dietary intake was collected and processed using the automated ASA24 online program, a validated tool that asks questions about food and beverage intake over the previous 24 h [42]. It details cooking methods, added ingredients, and supplements. It provides a comprehensive output of the participant’s dietary components including total calories, protein, carbohydrates, fiber, fat, caffeine, and iron. Data were included if the participant completed a minimum of two out of three total recalls and the results were averaged across all included days for analyses [47]. All participants (regardless of physical activity levels) were then grouped based on this dietary data. Those who met protein recommendations (≥1.1 g protein intake per kg body weight per day) were compared to those who did not. Those who met fiber recommendations (≥25 g fiber intake/day) were then separately compared to those who did not.

- Statistical analysis

Data were analyzed using IBM SPSS Statistics for Macintosh, Version 28.0, Armonk, NJ: IBM Corp. All data were analyzed descriptively with Shapiro–Wilk tests of normality and checks for assumptions of proposed statistical analyses. Between-subjects ANOVAs (F statistic) were conducted to compare PA, protein, and fiber group differences in actigraph-derived sleep variables. Mann–Whitney U (U = test statistic, Z = standard deviation from the mean) tests were used to compare subjective sleep variables as these were not normally distributed. Spearman’s rho correlations were run as a secondary analyses to examine the relationship between physical activity, dietary, and sleep variables.

## 3. Results

### Demographics/Covariates

**Figure 1:**
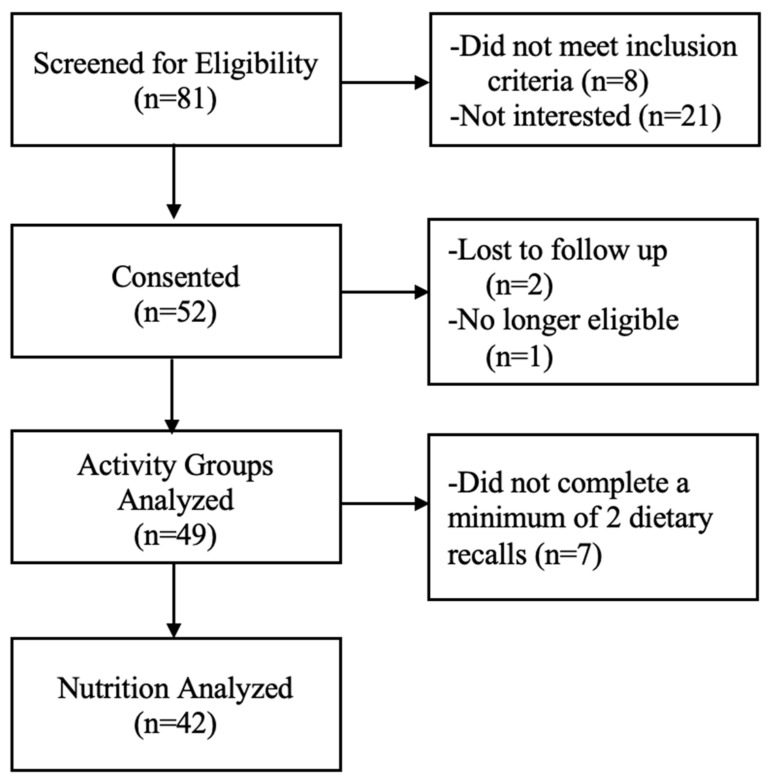
Consort flow diagram.

A total of 49 pregnant participants aged 31.9 ± 4.1 years old (Table 1) completed the study and were included in the physical activity analyses; 42 were included in the nutrition analyses (Figure 1). There were no differences between physical activity, protein, or fiber groups in age, race, socioeconomic status, or pre-pregnancy BMI (*p*s > 0.05) except for fiber groups which differed by age. Women who met fiber guidelines were older than women who did not meet fiber guidelines (*p* = 0.015).

Participants who met physical activity guidelines reported a lower global PSQI score (which indicates better sleep quality, U = 200.5, Z = −1.99, *p* = 0.049) and a lower PSQI sleep latency component score (which indicates falling asleep faster, U = 178.0, Z = −2.62, *p* = 0.01) (Table 2) compared to participants who did not. Poor sleep quality was common among participants, with 69% of NPA participants and 52% of PA participants exhibiting a PSQI global score >5; however, the prevalence did not differ between groups (*p* > 0.05). Self-reported daytime sleepiness did not differ between groups (*p* > 0.05), and prevalence of moderate-to-severe daytime sleepiness did not differ between groups (NPA: 40%, PA: 26%, *p* > 0.05). There were no differences between physical activity groups on objective sleep measures, including bedtime, waketime, sleep period, efficiency, WASO, total sleep time and fragmentation (Table 2; *ps* > 0.05). Among all participants, those with more MET minutes of MVPA per week reported a lower global PSQI score (Spearman’s rho (37) = −0.33, *p* = 0.02), and exhibited, via actigraphy, an earlier waketime (Spearman’s rho = −0.29, *p* = 0.04).

Participants who consumed the recommended daily amount of protein (1.1 g/kg body weight, n = 26) exhibited higher sleep efficiency (F(1, 40) = 6.19, *p* = 0.017), less WASO (F(1, 40) = 4.20, *p* = 0.047), longer total sleep time (F(1, 40) = 4.42, *p* = 0.042), and a marginally earlier bedtime (F(1, 40) = 3.68, *p* = 0.062; Table 3). There were no differences between groups for subjective sleep quality (PSQI Global, *p*s > 0.05) or daytime sleepiness (ESS; *p*s > 0.05). Covarying for age, participants who consumed the recommended daily amount of fiber (25 g/day, n = 16) exhibited greater fragmentation (F(1, 40) = 3.99, *p* = 0.017) compared to those who did not but the groups did not differ on any other objective measure of sleep (*p*s > 0.05). There were no differences between fiber groups for subjective sleep quality (PSQI Global, *p*s > 0.05) or daytime sleepiness (*p*s > 0.05; Table 4).

Among all participants, greater protein intake (%kcal) marginally associated with a lower PSQI global score (Spearman’s rho = −0.29, *p* = 0.063), particularly derived from the sleep duration component (Spearman’s rho = −0.38, *p* = 0.012), whereas carbohydrate and fat intake (%kcal) were not related with subjective measures. Among all participants, lower carbohydrate intake (%kcal) and greater fat intake (%kcal) associated with longer sleep period (carb: rho = −0.36, *p* = 0.02, fat: rho = 0.32, *p* = 0.04) and total sleep time (carb: Spearman’s rho = −0.36, *p* = 0.019); fat: rho = 0.32, *p* = 0.038). Greater fiber associated with an earlier bedtime (Spearman’s rho = −0.33, *p* = 0.032). Saturated fat was not associated with objective or subjective sleep outcomes (*p*s > 0.05).

## 4. Discussion

The objective of this study was to investigate if meeting existing guidelines related to physical activity and diet impacted sleep in pregnancy. Women who met physical activity guidelines reported better subjective sleep quality and reported fewer issues with falling asleep compared to women who did not. However, there were no differences between physical activity guideline groups on objective measures of sleep. Women who met protein dietary guidelines exhibited better sleep efficiency, less wake-after sleep onset, and more total sleep time compared to women who did not. However, there were no differences between protein guideline groups on subjective measures of sleep. Thus, nutrition and physical activity may have different effects on sleep and both should be addressed for optimal subjective and objective sleep outcomes.

Our finding that greater MVPA associates with improved subjective sleep quality is consistent with findings from several randomized control trials that observed beneficial effects of exercise interventions during pregnancy on the PSQI global score [18,48,49]. Furthermore, our correlational findings show that more minutes of MVPA associate with progressive improvements in sleep quality, suggesting that getting pregnant women to engage in any additional amount of moderate-to-vigorous intensity exercise could confer benefits for perceptions of sleep quality. There are a few proposed mechanisms for the benefits of exercise on sleep. Exercise is a stressor that activates the sympathetic nervous system and temporally increases heart rate [50]. Regular exercisers benefit from a reduction in resting heart rate due to modulation of the sympathetic nervous system which can attenuate physiological responses to stress and positively impact sleep [50]. Regular exercise has also been found to be beneficial for sleep through improvements in mood states and fatigue perception [51]. Interventions using resistance exercise during pregnancy found improvements in both physical and mental fatigue [52] along with a preservation of quality of life throughout pregnancy [53]. Future research should further investigate the relationship between exercise and subjective sleep to better determine the underlying mechanisms.

The benefits of protein for sleep during pregnancy has not been previously studied and the evidence among non-pregnant adults is mixed [24,54], with a meta-analysis finding no relationship between sleep and protein (>1 g/kg/day of protein or ≥25% kcal) whereas another study observed a positive association between protein (%kcal) and sleep in middle and older aged individuals. Furthermore, a cross sectional study found a positive association between protein intake and/or regular exercise with sleep quality [55]. Given that caloric demand increases and protein intake recommendations are higher during pregnancy, it is difficult to translate information from non-pregnant adults to pregnant women. It is possible that the relationship between protein and sleep is specific, or more pronounced in pregnancy, and future studies are needed with larger sample sizes to examine how protein intake may interact with exercise to impact sleep.

In women, there is often a lack of congruency between subjective and objective measures of sleep and these differences tend to be exacerbated during transitional phases (puberty, pregnancy, and perimenopause) [56]. In a study examining sleep between pre, peri, and postmenopausal women, subjective sleep reporting did not match objective polysomnography measures that found perimenopausal women had a greater percentage of deep sleep than premenopausal women but reported more sleep dissatisfaction [57]. The underlying cause between the observed differences has not been determined, but future research should incorporate both types of measurements to provide a more complete depiction of sleep in pregnancy. This is particularly important given our findings that different lifestyle interventions may have effects on one type of sleep outcome and not another. It is important to note that both measures of sleep, the perceptions of one’s sleep as well as the actual sleep behavior/physiology, are critical for health and wellbeing.

In this sample of pregnant women who were in gestational weeks 28–34, well educated, and of higher SES, only n = 10 met dietary guidelines for both protein and fiber and population studies show that the majority of pregnant women do not meet physical activity, protein, or fiber guidelines [20,34,36]. Thus, interventions to help women meet these basic physical activity and healthy diet criteria are critically needed. Hawkins et al. investigated a lifestyle intervention that included both physical activity and dietary guidance during the third trimester of pregnancy and postpartum period in women at risk for developing Type 2 diabetes [58]. The women randomized to the lifestyle intervention had a better change in subjective sleep quality over the course of the post-partum period compared to the control group [59]. The amount of exercise, quantity of calories, and type of dietary intervention were not reported but the goal of the study was to reduce postpartum weight retention. Given that both physical activity and pregnancy require additional caloric intake, it may be more helpful to focus on increasing protein consumption and promoting fruit and vegetable intake rather than focusing on weight management, to help pregnant women without impacting fetal growth.

There are a few limitations in this study. First, the study was conducted in a small sample of women during the early third trimester and may not be generalizable to the entire pregnancy. Sleep, physical activity, and dietary patterns are particularly dynamic during pregnancy and have varying underlying causes (i.e., hormonal fluctuations in the first trimester vs. anatomical changes in the third trimester) [22]. Future studies are needed to determine the relationship between these health behaviors at varying stages of pregnancy. Second, self report measures were utilized for the dietary analysis and are limited by a participant’s ability to properly recall and quantify every aspect of their food intake. Also, our dietary analyses were limited to macronutrient consumption. When we attempted to examine micronutrient intake, we noted significant ranges in reporting that suggested some women included intake from their prenatal vitamin whereas other women did not. Future work is needed to better understand how micronutrients, such as folic acid and tryptophan, are associated with sleep outcomes. Finally, this sample of women does not reflect the general population in terms of race and socioeconomic status. Given that socioeconomically disadvantaged women are at higher risk for sleep disturbances and less likely to meet physical activity and dietary guidelines [60], future work is needed to understand the relationship between these health variables in this vulnerable group.

## 5. Conclusions

Physical activity and macronutrient composition both impact sleep during pregnancy, with exercise more associated with subjective measures and diet more associated with objective measures. Pregnant women should be advised to incorporate more minutes of MVPA into their daily routine to improve subjective sleep quality and to meet protein guidelines and intake a diet higher in fat and lower in carbohydrates to improve objective sleep outcomes.

## Figures and Tables

**Table 1:** Participant demographics.

Variable	Average ± SD or (N)
Age	31.9 ± 4.1 years
Pre-Pregnancy BMI	23.9 ± 4.1
Parity	
Nulliparous	26
Multiparous	23
Race	
Asian	3
Black or African American	2
White or Caucasian	37
Hispanic/Latinx	6
Other	1
Education	
Grade 12 or GED (high school graduate)	1
1–3 years after high school or technical school	4
4 years or more (college graduate)	15
Advanced degree	28
Prefer not to answer	1
Household income	
<$25,000	1
$25,000–49,999	1
$50,000–74,999	6
$75,000–99,999	2
$100,000–149,999	10
$150,000–199,999	17
>$200,000	6
Prefer not to answer	6
Employment	
Employed	40
Not employed	9
Marital status	
Married	42
Divorced	0
Separated	1
Never married	2
Living with partner	2
Prefer not to answer	2
Nutrition	
Total kcal	2229.3 ± 637.9
Carbohydrates (g)	249.8 ± 81.2
Total Fats (g)	96.1 ± 38.0
Total Protein (g)	91.3 ± 30.6
Fiber (g)	22.4 ± 9.4
Diet composition	
Percent carbohydrates	45.2 ± 8.6%
Percent fats	38.2 ± 7.7%
Percent protein	16.7 ± 4.3%

**Table 2:** Sleep outcomes between physical activity groups.

Outcome	NPA (n = 26)	PA (n = 23)	Significance
Actigraphy			
Duration	478.2 ± 51.6 min	482.8 ± 53.6 min	0.76
Efficiency	86.6 ± 5.6%	87.5 ± 5.0%	0.54
WASO	53.2 ± 19.4 min	53.0 ± 21.4 min	0.98
Fragmentation	30.6 ± 9.2	31.8 ± 9.8	0.66
Sleep time	424.6 ± 55 min	428.8 min	0.79
Bed time	10:48 p.m. ± 80 min	10:32 p.m. ± 82 min	0.5
Wake time	6:59 a.m. ± 59 min	6:39 a.m. ± 67 min	0.27
PSQI			
Global score	7.3 ± 3.5	5.3 ± 2.7	0.05 *
PSQI poor sleep (>5)	18 (69.2)%	12 (52.2)%	0.22
ESS	7.7 ± 4.6	6.4 ± 3.5	0.39
Moderate sleepiness (>10)	10 (40.0)%	6 (26.1)%	0.31

* Denotes statistical significance.

**Table 3:** Sleep outcomes between protein groups.

Outcome	Insufficient Protein (n = 16)	Meets Protein (n = 26)	Significance
Actigraphy			
Duration	465.6 ± 41.5 min	486.3 ± 52.2 min	0.19
Efficiency	84.3 ± 5.5%	88.3 ± 4.7%	0.02 *
WASO	60.0 ± 20.7 min	48.0 ± 18.4 min	0.05 *
Fragmentation	33.3 ± 9.0	28.9 ± 8.6	0.12
Sleep time	404.1 ± 50.2 min	437.7 ± 50.2 min	0.04 *
Bed time	11:11 p.m. ± 78 min	10:26 p.m. ± 72 min	0.06
Wake time	6:59 a.m. ± 67 min	6:38 a.m. ± 65 min	0.27
PSQI			
Global score	6.9 ± 3.5	6.5 ± 3.2	0.82
PSQI poor sleep (>5)	10 (62.5)%	18 (69.2)%	0.65
ESS	6.6 ± 4.2	7.5 ± 4.1	0.54
Moderate sleepiness (>10)	5 (31.3)%	9 (34.6)%	0.75

* Denotes statistical significance

**Table 4:** Sleep outcomes between fiber groups.

Outcome	Insufficient Fiber (n = 26)	Meets Fiber (n = 16)	Significance
Actigraphy			
Duration	483.0 ± 46.9 min	470.9 ± 52.7 min	0.29
Efficiency	87.3 ± 5.6%	86.0 ± 4.9%	0.09
WASO	51.4 ± 20.2 min	55.2 ± 20.3 min	0.22
Fragmentation	28.5 ± 8.4	34.0 ± 8.9	0.02 *
Sleep time	430.8 ± 48.8 min	415.3 ± 57.7 min	0.15
Bed time	10:50 p.m. ± 87 min	10:42 p.m. ± 59 min	0.92
Wake time	7:01 a.m. ± 68 min	6:24 a.m. ± 57 min	0.28
PSQI			
Global score	6.5 ± 3.5	6.9 ± 2.9	0.45
PSQI poor sleep (>5)	16 (61.5%)	12 (75.0%)	0.37
ESS	6.3 ± 4.0	8.5 ± 3.9	0.12
Moderate sleepiness (>10)	7 (26.9%)	7(43.8%)	0.3

*Denotes statistical significance

## Data Availability

The data presented in this study are available on request from the corresponding author. The data are not publicly available due to participant confidentiality.
